# Effects of a healthy lifestyle intervention and COVID-19-adjusted training curriculum on firefighter recruits

**DOI:** 10.1038/s41598-022-10979-2

**Published:** 2022-06-23

**Authors:** Fan-Yun Lan, Christopher Scheibler, Maria Soledad Hershey, Juan Luis Romero-Cabrera, Gabriel C. Gaviola, Ioanna Yiannakou, Alejandro Fernandez-Montero, Costas A. Christophi, David C. Christiani, Mercedes Sotos-Prieto, Stefanos N. Kales

**Affiliations:** 1 Department of Environmental Health, Harvard University T.H Chan School of Public Health, Boston, MA USA; 2 Department of Occupational Medicine, Cambridge Health Alliance, Harvard Medical School, Macht Building 427, 1493 Cambridge Street, Cambridge, MA 02139 USA; 3 Department of Occupational and Environmental Medicine, National Cheng Kung University Hospital, College of Medicine, National Cheng Kung University, Tainan, Taiwan; 4 Department of Internal Medicine, Hospital Universitario Reina Sofia, Córdoba, Spain; 5 Program in Biomedical Sciences, Boston University School of Medicine, Boston, MA USA; 6 Department of Occupational Medicine, Clínica Universidad de Navarra, Pamplona, Navarra Spain; 7 Instituto de Investigación Sanitaria de Navarra (IdiSNA), Pamplona, Navarra Spain; 8 Cyprus International Institute for Environmental and Public Health, Cyprus University of Technology, Limassol, Cyprus; 9 Department of Preventive Medicine and Public Health, School of Medicine, Universidad Autónoma de Madrid and CIBERESP (CIBER of Epidemiology and Public Health), Madrid, Spain; 10 IMDEA-Food Institute, CEI UAM+CSIC, Madrid, Spain

**Keywords:** Disease prevention, Occupational health, Public health

## Abstract

There are knowledge gaps regarding healthy lifestyle (HLS) interventions in fire academy settings and also concerning the impacts of the pandemic on training. We enrolled fire recruits from two fire academies (A and B) in New England in early 2019 as the historical control group, and recruits from academies in New England (B) and Florida (C), respectively, during the pandemic as the intervention group. The three academies have similar training environments and curricula. The exposures of interest were a combination of (1) an HLS intervention and (2) impacts of the pandemic on training curricula and environs (i.e. social distancing, masking, reduced class size, etc.). We examined the health/fitness changes throughout training. The follow-up rate was 78%, leaving 92 recruits in the historical control group and 55 in the intervention group. The results show an HLS intervention improved the effects of fire academy training on recruits healthy behaviors (MEDI-lifestyle score, 0.5 ± 1.4 vs. − 0.3 ± 1.7), systolic blood pressure (− 7.2 ± 10.0 vs. 2.9 ± 12.9 mmHg), and mental health (Beck Depression score, − 0.45 ± 1.14 vs. − 0.01 ± 1.05) (all P < 0.05). The associations remained significant after multivariable adjustments. Moreover, a 1-point MEDI-lifestyle increment during academy training is associated with about 2% decrement in blood pressures over time, after multivariable adjustments (P < 0.05). Nonetheless, the impacts of pandemic restrictions on academy procedures compromised physical fitness training, namely in percent body fat, push-ups, and pull-ups.

## Introduction

Accumulating evidence shows firefighting can lead to not only acute accidents and injuries^1,2^, but also to chronic disease^3–5^. Occupational hazards such as chemicals, extreme temperature, particles, and heavy physical demands due to firefighting have been widely investigated^6,7^, while emerging evidence discloses an unhealthy nutritional environment and sedentary behavior, which may contribute to chronic lifestyle-related diseases that are prevalent among firefighters^3,8,9^, including cancers, obesity, hypertension, cardiomegaly, coronary heart disease and other cardiovascular disease (CVD)^4,5,8,10^.

Physical fitness is of great interest in public safety workforces. Positive associations have been observed between physical fitness and training performance, lower occupational injury rates, decreased risk of subsequent CVD events, and decreased mortality^11–16^. Despite physical fitness requirements during academy training^17^, most fire departments do not require ongoing fitness training and most firefighters are obese/ overweight^18^. Studies have shown fire academy training does improve fire recruits’ health and fitness, but most of the benefits are lost soon after recruits become probationary firefighters^17,19^. A work environment that includes long sedentary periods, inadequate fitness requirements, insufficient and interrupted sleep, and a culture that may encourage unhealthy diets contributes to obesity, substandard fitness and chronic disease risks^9^.

Various preventive strategies and interventions have been attempted. Accumulating exercise interventions and physical training programs conducted among firefighters show positive effects on improving body composition, aerobic capacity, muscle power, endurance, and strength^20,21^. Other health promotion interventions including nutritional instruction and psychological resilience training also have beneficial effects on career firefighters’ body mass index (BMI), health behaviors, and mental health^22–24^.

Fire recruits are future firefighters who undergo academy training to be equipped with the professional skills and functional capacities needed for fire service. In the USA, fire academy training generally ranges between 12 and 20 weeks for career firefighters^9^. While existing evidence shows that academy training improves fire recruits’ physical fitness^17^, our previous study demonstrated that recruits’ blood pressures, both systolic and diastolic, increased by more than 2 mmHg throughout the training on average, despite other improved health parameters^19^. In addition, in the same study, we found that traditional academy training did not improve recruits’ dietary behaviors^19^. To our knowledge, while quite a few intervention studies on career firefighters have been conducted^20–24^, there is no existing health behavior/ lifestyle intervention research on fire recruits, although evidence has shown that early health education does have long-lasting health benefits^25^ and a healthy lifestyle (HLS) can be a potential solution for firefighters’ health concerns^9^.

Since March 2020, the COVID-19 pandemic has affected millions of workers all around the world^26^. While mitigation policies such as lockdowns and remote work have been implemented, they are not applicable to essential occupations, including training new firefighters^27^. Previous literature has demonstrated the risk of cluster transmission of SARS-CoV-2 (the virus causing COVID-19) infection among fire academies and its associated mental health impacts^28^. In fact, as fire academies needed to keep running during the pandemic, some adjustments to the existing training curriculum have been adopted to comply with public health regulations, such as mandated masking and increased social distancing. These adjustments include limiting class size, canceling large-group activities, conforming to social distancing during physical training, etc. Little is known about whether such adjustments influence the quality of academy training and its associated effects on recruits’ health and fitness. Therefore, we conducted this study to investigate the effects of (1) a healthy lifestyle (HLS) intervention and (2) an adjusted training curriculum due to the COVID-19 pandemic, by comparing the recruits from intervened academy classes to historical controls.

## Methods

### Study design and study population

In this time-controlled intervention study, a historical control group was used to evaluate the effects of the interventions. Therefore, two fire recruit populations were enrolled; (a) the control group comprised of two classes of recruits going through the academy training with existing, pre-pandemic training curricula and (b) the intervention group consisted of two classes of recruits enrolled during the pandemic and also receiving an HLS intervention.

The control group was recruited in early 2019 from two fire academies (academy A and B) in the New England area^19,29^. Both academies provide a 15–16 week training program that meets National Fire Protection Association (NFPA)’s standards, NFPA 1001: Standard for Fire Fighter Professional Qualifications. Except for minor differences between the two (for example, academy B requires recruits to stay overnight in the academy on training days while recruits at academy A go home every day after training, and academy B provides additional aquatic classes as part of their physical training), the recruits comprising the historical control group across academies were comparable according to our previous^19^ and current studies.

For the intervention group, we enrolled fire recruits from one fire academy in New England (academy B) and one in Florida (academy C) in late 2020. The training in academy C resembles that in academy B, regarding NFPA standards and overnight staying requirement, with similar training durations of 15 and 13 weeks for academies B and C, respectively.

All enrolled fire recruits who were older than 18 years old and provided informed consent were included. Those who did not consent to participate in the study or lacking essential demographic information (i.e., age and sex) were excluded. The current study is part of the “*Fire Recruit Health Study*” approved by the Institutional Review Board of Harvard T.H. Chan School of Public Health (IRB18-1902). We followed the Declaration of Helsinki throughout the study.

### Selected health outcomes

The outcomes selected for study included body composition, blood pressure, physical fitness testing, mental health screens, and lifestyle behaviors. All academies, except academy C, had complete data collection, while academy C provided only subjective outcomes (i.e. questionnaire). The related measurements were described in our previous studies^19,29^ and are summarized below.

Recruits’ BMI and percent body fat were examined as body composition outcomes. A clinic stadiometer (Portable Stadiometer 213, SECA, Hamburg, Germany) and a Bioelectrical Impedance Analysis scale (BIA) (BC-418 Segmental Body Composition, Tanita, Tokyo, Japan or InBody 230, Seoul, South Korea) using athletic mode, operated by experienced physical trainers or medical personnel, were used to retrieve the parameters. The measurements were performed at entry to the academy, mid-training (i.e. 8th week for academy A and 7th week for academy B), and academy graduation. Body composition data were not available at academy C.

Blood pressures were measured using an automated and calibrated sphygmomanometer (10 series, Omron, Kyoto, Japan), following professional guidelines^30^. The measurements were done before recruits started their daily training or during rest break. Each recruit was asked to rest seated for at least 5 min before being measured in a sitting position. The automated sphygmomanometer would then take three readings, with 1-min interval between each, and record an average. Blood pressures measurements were conducted at entry to academy and graduation, and were not available at academy C.

Select physical fitness outcomes were push-ups, pull-ups, and 1.5-mile running time, with each measurement taken at entry to academy, mid-training, and at graduation. These are existing tests used by the academies to evaluate recruits’ physical performance over time. Push-ups were determined as the number a recruit performed continuously in one minute, without breaking the cadence. Pull-ups were counted as the number in single trial with good cadence and overhead grip. Running time for 1.5 miles was recorded in minutes. Physical fitness testing results were not available at academy C.

We used a questionnaire to examine participants’ mental health and lifestyle behaviors, administered at their entry to the academy and at graduation. The questionnaire was comprised of components derived from validated questionnaires, incorporating a modified version of Beck Depression Inventory for Primary Care (BDI-PC) (total scoring 0–18)^31^, Patient Health Questionnaire (PHQ-9) (total scoring 0–27)^32^, and a modified version of Posttraumatic Stress Disorder Checklist (PCL-5) (total scoring 0–76)^33^, with higher scores indicating worse mental health. As to lifestyle behaviors, the questionnaire contained items needed to calculate the MEDI-lifestyle score^29^, which is a 7-item healthy lifestyle score ranging from 0 to 7, embodying BMI, smoking history, dietary pattern (measured by the PREDIMED score, a 14-item Mediterranean Diet adherence screener^34^), physical activity^35^, sedentary behavior (measured by time spent watching television), daily sleep time, and afternoon naps. In particular, one point was given for each of the following: no smoking in the last 6 months, physical activity equivalent to greater than 16 METs-h/wk, PREDIMED score more than or equal to nine points, BMI less than or equal to 30 kg/m^2^, TV screen time less than 2 h/day, sleeping time between 7 and 8 h/day, and taking daytime naps; otherwise a value of 0 would be given to each item.

### Interventions

Compared with the historical control group, the intervened classes underwent the following changes in the existing training materials implemented by the academies.

First, the academies adopted an HLS intervention throughout the 13- or 15-week training based on the firefighters’ Mediterranean pyramid^24^, which illustrates a healthy lifestyle combination of balanced nutrition, regular physical activity, restorative sleep, positive social and family connections with resiliency strategies, and the avoidance of tobacco and other toxic substances. Each participant was given (a) access to a web-based toolkit (https://www.hsph.harvard.edu/firefighters-study/feeding-americas-bravest/) that includes information and resources for “Survival Mediterranean Style”, (b) a half-hour talk on healthy lifestyle at the entry to the academy training, (c) a waterproof, plastic paper sheet illustrating the firefighters’ Mediterranean pyramid, (d) a refrigerator magnet with the Mediterranean pyramid on it, (e) weekly nutrition/lifestyle tips throughout the academy training, and (f) an introduction to meditation/breathing exercise apps (for example, the Calm app (San Francisco, USA)). Except for (e), all intervention materials were given at the beginning of the academy training. The participants were able to review the HLS contents via the provided measures throughout the training period. While the practice of the HLS is voluntary, sponsored olive oil was supplied to the central kitchen of academy B and consumed by the fire recruits when they stayed at the academy on weekdays, and academy C gave each recruit of the intervened class a WHOOP (Boston, USA) wearable device that tracked recruits’ fitness and physiological parameters. Notably, weekly homework such as practicing a healthy recipe was assigned along with the weekly tips to the recruits. With the collaboration with the academies, extra training credits were given as incentives if the recruits showed their adherence to the HLS outside regular training time.

Second, as the intervention classes were trained during the COVID-19 pandemic, some curriculum adjustments were made to conform to public health policies. These changes included face masking required at all times during the training, limited class size, and shifting large group activities (such as group running) to small group physical training to increase social distancing. Moreover, previously there was a weekly 1-h aquatic training in the swimming pool at academy B, but since the pool was closed, the aquatic classes were replaced by weekly 1-h joint mobility exercise, in which recruits conducted a whole-body slow paced, yoga-like stretching workout.

### Statistical analysis

Baseline characteristics and select health outcomes were reported as mean ± standard deviation or median (Q1-Q3) for continuous variables after checking for normality, or number (%) for categorical variables, and compared between groups using the t-test or the Wilcoxon rank sum test, as appropriate, for continuous variables and the Pearson's Chi-squared test with Yates' continuity correction or the Fisher’s exact test, as appropriate, for categorical variables.

Furthermore, we computed the changes in select health outcomes over time during academy training by calculating the longitudinal difference “the measurement at graduation—the measurement at baseline”, and presenting them as mean ± standard deviation, after checking normality. The differences in temporal changes between the intervention group and the control group were compared using the t-test.

For multivariable adjustment, mixed effects models were built incorporating the interaction term “Intervention Group × Time” to examine whether the health changes over time during academy training differed between the two groups. Potential confounders based on our domain knowledge and the baseline characteristics comparisons were included into the models. These are age, sex, baseline percent body fat, baseline push-ups, and/or baseline BDI-PC scores.

Finally, we built multivariable adjusted linear models to regress the health changes on the change of MEDI-lifestyle score, in order to demonstrate the changes in health per unit changes of MEDI-lifestyle score. For these models, the health changes throughout training were defined as percent changes from baseline measurements, except for those variables with any values of zero at baseline (i.e. pull-ups, BDI-PC, PCL-5, and PHQ-9).

All P values reported are two-tailed and a P < 0.05 was considered statistically significant. We used the R software (version 3.6.3) to conduct the statistical analyses.

### Sensitivity analysis

With regard to the differences in training across academies, we conducted further sensitivity analysis limiting to fire recruits at the academy with both historical control class and intervention class available, which is academy B. In fact, there was one more class at academy B that took place in early 2020, receiving the lifestyle intervention, but undergoing unexpected training interruption for 3 months due to the initial COVID-19 outbreak. By comparing the three classes at academy B (i.e. the historical control class, COVID-19 interrupted class, and the intervention class), we were able to examine the effects of the intervention as well as the impact of the 3-month training interruption on recruits’ health. Notably, only the body composition and fitness testing data are available for the COVID-19 interrupted class.

In addition, while there were differences in the intervention contents across the academies B and C, as described above, we further conducted secondary analyses to investigate if the health changes differed between the two populations (i.e., the fire recruits comprising the intervention group from academy B and C, respectively) throughout academy training. Since objective data were not available at academy C, only subjective measurements (i.e., behavioral and mental health outcomes) could be compared.

### Ethics approval and consent to participate

The study is part of the “*Fire Recruit Health Study*”, which was approved by the Institutional Review Board of Harvard T.H. Chan School of Public Health (IRB18-1902), and we followed the Declaration of Helsinki throughout the study.

## Results

A total of 172 recruits were eligible for the main analyses, including 101 in the historical control group and 71 in the intervention group. Among them, 95 (94.1%) from the control group and 69 (97.2%) from the intervention group consented to participate. After excluding those with missing essential data (i.e. age and sex) and those failing to complete the academy training, 92 (91.1%) in the control arm, comprised of 56 from academy A and 36 from academy B, and 55 (77.5%) in the intervention arm, comprised of 37 from academy B and 18 from academy C, were included in the analyses.

There was no significant difference in recruits’ age (28.6 ± 5.1 years vs. 29.3 ± 5.2 years, P = 0.428) and sex (96.7% male vs. 100.0% male, P = 0.293) between the two groups, while the control group showed lower baseline percent body fat (20.5 ± 6.9% vs. 23.9 ± 7.2%, P = 0.013) and performed more baseline push-ups (37.7 ± 14.1 vs. 28.7 ± 10.9, P < 0.001) than the intervention group. Moreover, the intervention group reported more depressive symptoms (i.e. higher BDI-PC score) at baseline (BDI-PC IQR: 0–1 vs. 0–0, P = 0.013) (Table 1).

**Table 1:** Baseline demographics, body composition, physical fitness, and mental health of the recruits of the two groups.

	Historical control group	Intervention group	*P* value comparing recruits of the two groups
N	92	55	
Age	28.6 ± 5.1	29.3 ± 5.2	0.428
Male N (%)	89 (96.7%)	55 (100.0%)	0.293^a^
BMI (kg/m^2^)	27.9 ± 3.8	28.5 ± 4.2	0.484
%BF	20.5 ± 6.9	23.9 ± 7.2	0.013
Systolic BP (mmHg)	124.9 ± 10.8	125.3 ± 7.5	0.846
Diastolic BP (mmHg)	71.1 ± 9.4	74.3 ± 7.6	0.067
Push-ups	37.7 ± 14.1	28.7 ± 10.9	< 0.001
Pull-ups	7.4 ± 5.3	6.7 ± 5.2	0.480
Run time 1.5 miles (minutes)	12.5 ± 1.8	12.3 ± 2.2	0.554
PREDIMED score	6.7 ± 2.0	6.7 ± 2.3	0.967
MEDI-lifestyle score	4.0 ± 1.1	4.0 ± 1.1	0.697
Beck questionnaire	0 (0–0)	0 (0–1)	0.013^b^
PCL questionnaire	3 (0–8)	2 (1–7)	0.920^b^
PHQ score	0 (0–2)	0.5 (0–2)	0.932^b^

Median (Q1–Q3) for Beck questionnaire, PCL questionnaire, and PHQ score. Mean ± SD for other variables except for Male. BMI, %BF, blood pressures, push-ups, pull-ups, run time, and MEDI-lifestyle score are only available for one academy of the intervention group (n = 37).

*BMI* body mass index, *%BF* percent body fat, *BP* blood pressure, *Beck* Beck depression inventory for primary care, *PCL* post-traumatic stress disorder (PTSD) questionnaire, *PHQ* patient health questionnaire.

^a^Derived from Fisher’s exact test.

^b^Derived from Wilcoxon rank sum test with continuity correction.

**Figure 1:**
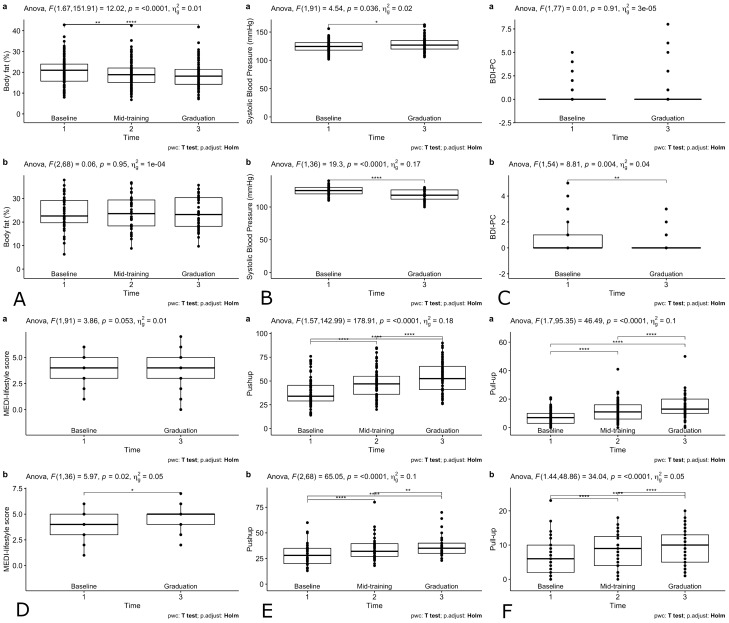
Box plots showing the distributions of recruits’ selected health profiles at baseline, mid-training, and academy graduation comparing (**a**) the historical control group (n = 92) and (**b**) the intervention group (n = 55). A. Body fat; B. systolic blood pressure; C. BDI-PC (Beck Depression Inventory for Primary Care); D. MEDI-lifestyle score; E. push-ups; F. pull-ups. The plots were derived from the recruits whose data were available at each time point. Post-hoc paired analysis with Holm adjustments were demonstrated by *P < 0.05, **P < 0.01, ***P < 0.001, ****P < 0.0001. Body fat, systolic blood pressure, push-ups, pull-ups, and MEDI-lifestyle score are only available for one academy of the intervention group (n = 37).

Table 2 demonstrates the select outcome changes over time during academy training. While both groups show decreased percent body fat and increased push-ups and pull-ups throughout training, less improvement is observed in the intervention group (percent body fat change: − 0.2 ± 2.8% vs. − 1.8 ± 3.5%, P = 0.013; push-ups change: 8.2 ± 4.9 vs. 16.8 ± 10.4, P < 0.001; pull-ups change: 2.8 ± 2.5 vs. 5.8 ± 5.1, P = 0.001). On the other hand, the intervention group reported more lifestyle improvement than the control (MEDI-lifestyle score change: 0.5 ± 1.4 vs. − 0.3 ± 1.7, P = 0.005), and a greater decrease in depressive symptoms (BDI-PC score change: − 0.45 ± 1.14 vs. − 0.01 ± 1.05, P = 0.023). In addition, systolic blood pressure was lowered in the intervention group but got higher in the control group throughout academy training (− 7.2 ± 10.0 mmHg vs. 2.9 ± 12.9 mmHg, P < 0.001) (Table 2, Fig. 1).

**Table 2:** Changes in recruits’ selected body composition and physical fitness measurements from baseline to graduation of the two groups.

	Historical control group	Intervention group	*P* value comparing recruits of the two groups
N	92	55	
BMI (kg/m^2^)	0.02 ± 1.00	− 0.18 ± 0.86	0.302
%BF	− 1.8 ± 3.5	− 0.2 ± 2.8	0.013
Systolic BP (mmHg)	2.9 ± 12.9	− 7.2 ± 10.0	< 0.001
Diastolic BP (mmHg)	3.8 ± 10.1	3.3 ± 10.0	0.818
Push-ups	16.8 ± 10.4	8.2 ± 4.9	< 0.001
Pull-ups	5.8 ± 5.1	2.8 ± 2.5	0.001
Run time 1.5 miles (min)	− 1.3 ± 0.8	− 1.0 ± 0.9	0.093
MEDI-lifestyle sco﻿re	− 0.3 ± 1.7	0.5 ± 1.4	0.005
PREDIMED score	− 0.1 ± 2.0	0.3 ± 1.9	0.293
Physical activity rating	0.6 ± 1.5	0.9 ± 1.3	0.340
TV screening time (h/day)	− 0.7 ± 1.7	− 0.5 ± 1.7	0.445
Sleeping time (h/day)	0.01 ± 0.99	0.09 ± 1.06	0.664
Afternoon nap (time/week)	− 0.04 ± 1.13	− 0.30 ± 1.38	0.245
Beck questionnaire	− 0.01 ± 1.05	− 0.45 ± 1.14	0.023
PCL questionnaire	− 1.0 ± 7.6	− 1.4 ± 6.6	0.731
PHQ score	− 0.3 ± 2.6	− 0.5 ± 2.3	0.549

Mean ± SD. BMI, %BF, blood pressures, push-ups, pull-ups, run time, and MEDI-lifestyle score are only available for one academy of the intervention group (n = 37).

*BMI* body mass index, *%BF* percent body fat, *BP* blood pressure, *Beck* Beck depression inventory for primary care, *PCL* post-traumatic stress disorder (PTSD) questionnaire, *PHQ* patient health questionnaire.

The associations remained significant after multivariable adjustments. Table 3 shows that the intervention group had less improvement in percent body fat, push-ups, and pull-ups over time, but more improvements in MEDI-lifestyle score, systolic blood pressure, and BDI-PC questionnaire, after accounting for recruits’ age, sex, baseline percent body fat, baseline push-ups, and/or baseline BDI-PC score (all P < 0.05 for the interaction term “Intervention Group × Time”).

**Table 3:** Multivariable adjusted selected health profiles comparing the academy training effects on the intervention group (N = 55) to the historical control group (N = 92).

Outcome	Predictor	Beta coefficient	Standard error	*P* value
BMI (kg/m^2^)	Time	0.01	0.05	0.868
	Intervention group	− 0.70	0.51	0.170
	Intervention group × Time	− 0.10	0.09	0.300
%BF	Time	− 0.89	0.18	< 0.001
	Intervention group	2.03	1.48	0.174
	Intervention group × Time	0.81	0.34	0.016
Systolic BP (mmHg)	Time	− 0.01	0.06	0.888
	Intervention group	0.64	0.31	0.038
	Intervention group × Time	− 0.25	0.10	0.019
Diastolic BP (mmHg)	Time	1.88	0.52	< 0.001
	Intervention group	2.06	2.51	0.413
	Intervention group × Time	− 0.23	0.98	0.818
Push-ups	Time	8.41	0.48	< 0.001
	Intervention group	− 3.01	2.82	0.288
	Intervention group × Time	− 4.31	0.89	< 0.001
Pull-ups	Time	2.87	0.28	< 0.001
	Intervention group	3.65	0.86	< 0.001
	Intervention group × Time	− 1.48	0.45	0.001
Run time 1.5 miles (min)	Time	− 0.64	0.04	< 0.001
	Intervention group	− 0.84	0.36	0.020
	Intervention group × Time	0.15	0.08	0.074
MEDI-lifestyle score	Time	− 0.17	0.08	0.041
	Intervention group	− 0.08	0.29	0.778
	Intervention group × Time	0.44	0.15	0.005
PREDIMED score	Time	0.01	0.12	0.962
	Intervention group	0.41	0.53	0.440
	Intervention group × Time	0.24	0.21	0.253
Physical activity rating	Time	0.31	0.08	< 0.001
	Intervention group	− 0.24	0.37	0.529
	Intervention group × Time	0.21	0.15	0.142
TV screening time (h/d)	Time	− 0.37	0.09	< 0.001
	Intervention group	− 1.12	0.43	0.010
	Intervention group × Time	0.31	0.17	0.074
Sleeping time (h/day)	Time	− 0.004	0.057	0.951
	Intervention group	0.14	0.29	0.638
	Intervention group × Time	0.06	0.10	0.570
Afternoon nap (time/week)	Time	− 0.01	0.06	0.848
	Intervention group	0.07	0.31	0.813
	Intervention group × Time	− 0.19	0.11	0.091
Beck questionnaire	Time	− 0.003	0.059	0.953
	Intervention group	0.65	0.29	0.026
	Intervention group × Time	− 0.25	0.10	0.017
PCL questionnaire	Time	− 0.62	0.39	0.114
	Intervention group	− 2.80	1.47	0.060
	Intervention group × Time	0.50	0.68	0.463
PHQ score	Time	− 0.18	0.13	0.174
	Intervention group	− 1.33	0.57	0.021
	Intervention group × Time	0.21	0.23	0.364

Statistics derived from age, sex, baseline %BF, baseline push-ups, and/or baseline Beck score adjusted mixed effects models. BMI, %BF, blood pressures, push-ups, pull-ups, run time, and MEDI-lifestyle score are only available for one academy of the intervention group (n = 37).

*BMI* body mass index, *%BF* percent body fat, *BP* blood pressure, *Beck* Beck depression inventory for primary care, *PCL* post-traumatic stress disorder (PTSD) questionnaire, *PHQ* patient health questionnaire.

In order to examine whether the changes in health (i.e. the difference “the measurement at graduation—the measurement at baseline”) were related to the changes in healthy lifestyle, we pooled both groups together and built multivariable linear regression models with the same covariates as the mixed models used for Table 3, with additional adjustments for different academy classes (i.e. control group or intervention group) to account for intervention effects. The results show that a 1-point MEDI-lifestyle increment during academy training is associated with 1.95% and 2.73% decrements in systolic and diastolic blood pressure, respectively (both P < 0.05). We did not observe significant associations between the change in MEDI-lifestyle score and other select health outcomes (Table 4).

**Table 4:** Multivariable adjusted changes in selected health profiles per 1-point MEDI-lifestyle score increment during academy training among the whole fire recruit cohort (N = 129).

Change during academy training^a^	Beta coefficient	Standard error	*P* value
BMI	0.16	0.18	0.376
%BF	− 0.78	0.99	0.436
Systolic BP	− 1.95	0.55	< 0.001
Diastolic BP	− 2.73	0.84	0.002
Push-ups	− 1.33	2.42	0.581
Pull-ups	0.31	0.30	0.305
Run time 1.5 miles	0.10	0.32	0.767
Beck questionnaire	0.05	0.08	0.516
PCL questionnaire	− 0.27	0.53	0.617
PHQ score	− 0.14	0.17	0.419

Statistics derived from age, sex, baseline %BF, baseline push-ups, baseline Beck score, and academy class adjusted linear regression models. BMI, %BF, blood pressures, push-ups, pull-ups, run time, and MEDI-lifestyle score are only available for one academy of the intervention group (n = 37), leaving a total of 129 recruits (37 from the intervention group and 92 from the historical control group) for the analyses.

*BMI* body mass index, *%BF* percent body fat, *BP* blood pressure, *Beck* Beck depression inventory for primary care, *PCL* post-traumatic stress disorder (PTSD) questionnaire, *PHQ* patient health questionnaire.

^a^Absolute number changes for pull-ups, Beck, PCL, and PHQ. Percent changes for other variables.

In the sensitivity analyses limited to classes from academy B and adding one more class that received the healthy lifestyle intervention but sustained an unexpected 3-month training interruption due to the COVID-19 pandemic, we found no significant differences in the select outcomes when comparing the intervention class to the control. However, when comparing the COVID-19 interrupted class to the control, the recruits’ BMI decreased by 0.33 more per measurement time (i.e. baseline, mid-training, to graduation) than the control (P = 0.036) after multivariable adjustments. Nonetheless, the COVID-19 interrupted class shows an average of 1.3 fewer push-ups and 0.58 min additional running time for 1.5 mile run per measurement time than the control class (both P < 0.05 in the multivariable adjusted mixed effects models). When comparing the COVID-19 interrupted class with the intervention class, the difference in the 1.5 mile running time remained significant (0.58 min more per measurement time after multivariable adjustment, P < 0.001) (Supplemental Tables S1–S4, Supplemental Fig. S1).

For the secondary analyses comparing the health changes throughout academy training across academy B and C from the intervention group, there were no significant differences in the changes of PREDIMED score, physical activity, sleeping and napping behaviors, and the BDI-PC score. Nonetheless, those from the academy C showed more decreased TV screening time (− 1.21 ± 1.6 vs. − 0.14 ± 1.7 h/d, P = 0.029), more decreased PCL-5 score (− 4.3 ± 9.5 vs. − 0.2 ± 4.6, P = 0.045), and more decreased PHQ-9 score (− 1.7 ± 3.0 vs. 0.0 ± 1.5) throughout academy training.

## Discussion

In our present intervention study using a historical control group, we investigated the combined effects of (1) a healthy lifestyle intervention and (2) an adjusted training curriculum due to the COVID-19 pandemic on fire recruits’ health and fitness during academy training. Our novel findings show the MEDI-lifestyle score increased over time by 0.5 points more in the intervention group than the control group, and systolic blood pressure and self-reported depressive symptoms had greater decrements during the training. In fact, each MEDI-lifestyle score increment during the study period is associated with 1.95% and 2.73% decrement in systolic and diastolic blood pressure, respectively. Nonetheless, we also found that the intervention group had smaller reductions in body fat and had less push-ups/pull-ups improvement than the control group, which is likely due to the pandemic-required adjustments in the training curriculum.

To our knowledge, the current work is the first lifestyle intervention study in a fire academy setting, while unfortunately that could not be a pure lifestyle intervention but one impacted by unprecedented adjustments in the training curriculum due to the pandemic. It is biologically implausible to attribute the finding of increased MEDI-lifestyle scores over time to pandemic-related restrictions in the curriculum. Therefore, it is likely that the lifestyle intervention does improve recruits’ healthy lifestyle behaviors. In fact, a Swiss study investigating the effect of a healthy eating promotion program on career firefighters shows modest improvements on eating habits after one-year follow-up^36^, although the findings were limited by a lack of control group and a small sample size (n = 28). Another study on fourteen volunteer firefighters demonstrated that a 30-min health promotion educational intervention increased participants’ health literacy^37^. Accordingly, our results show that the intervened fire recruits improved their HLS behaviors, using a well-defined control group, a more intensive lifestyle intervention, and an adequate follow-up period.

Significant decrements in blood pressures among the intervention group is another novel finding of our present study. For the control group, recruits receiving the existing fire academy curriculum actually had increased blood pressures throughout the training, despite improved body composition and physical fitness^19^. Since hypertension and CVD are prevalent diseases in the fire service workforce^3,38^, it is important to implement vascular health and blood pressure interventions^39^. In fact, evidence has shown that a greater adherence to a healthy dietary pattern is associated with better cardiometabolic profiles^40,41^. In addition, our previous research on fire recruits also demonstrates a negative association between healthy lifestyle adherence and hypertension prevalence^29^. In agreement with the literature, we found that the healthy lifestyle intervention group had lowered blood pressures during academy training, and per one point increment in MEDI-lifestyle score throughout training is associated with around 2% decrement in both systolic and diastolic blood pressure, highlighting the effectiveness of the healthy lifestyle intervention.

We found in the present study that recruits attending academy training during the COVID-19 pandemic were more depressed at baseline. While the qualification/ entry exams for becoming a fire recruit remained the same, it is not surprising that living through the pandemic would be associated with an increase in recruits’ depressive symptoms at baseline. In fact, evidence has shown psychological distress is of great public health concern among the general population in the midst of the pandemic^42^. The fear of being infected, a sense of liberty deprivation due to quarantine and lock-down, being socially isolated, and worrying about transmission among family members could all contribute to the substantial psychological impacts^42–46^. Fortunately, research demonstrates that adopting a healthy lifestyle has benefits against depression. In an 8-year prospective study of 14,908 participants who were initially depression-free, an optimal healthy lifestyle adherence (a combination of the Mediterranean diet, increased physical activity, optimal sleep pattern, decreased sedentary period, no smoking, moderate drinking, optimal BMI, and positive social connections) was associated with a 32% lower risk of incident depression^47^. Accordingly, we found the lifestyle-intervened recruits had more improved depressive symptoms throughout academy training than the historical control group. In addition, the findings of our secondary analyses are indicative of the potential effectiveness of wearable devices on fire recruits’ behavioral and mental health, as those given wearable devices (i.e., those from academy C) reported more improvement in TV screening time, PCL-5, and PHQ-9 scores.

To our knowledge, there is no existing literature examining how pandemic-related adjustments in training curricula could affect physical fitness. In our current study, the intervention group shows suboptimal training effects on recruits’ body fat and pull-up/push-up capacities, and a 3-month training interruption compromised recruits’ aerobic fitness (i.e. 1.5-mile running) improvements. In fact, research on the general population has shown the physical distancing and lock-down policies generally reduce people’s physical activity and fitness^48,49^. Despite a lack of evidence on fire recruits, firefighters, or other emergency respondents, professional recommendations for competitive athletes’ training during the pandemic have been conservative, with lower intensity and more flexibility^50,51^. Since long-term detraining could decrease muscle mass, strength, and endurance^52^, the observed decreased aerobic function improvement among the interrupted class is not surprising. In addition, a study on military recruits after a COVID-19 cluster outbreak shows decreased fitness among affected personnel, compared with others receiving the same military training^53^. Accordingly, we found an adjusted academy training curriculum due to the pandemic improved recruits’ fitness less than the historical control group. This is likely because of conservative changes made to the training, reduced self-exercise during rest time, and possible subclinical/ asymptomatic transmission among recruits. Therefore, future research is warranted to see if the academy training effects and capacities on physical fitness will meet pre-pandemic levels as the COVID-related restrictions are getting relaxed and herd immunity is increasing (due to vaccination and prior infection).

The present study has several strengths. First, we adopted a time-controlled design, using a historical control group as the reference, which is the most rigorous study design regarding the research aims. While fire recruits undergo academy training in groups, randomization at the individual level is inappropriate because of potential spillover effects^54^. Historical academy classes that had the same entry qualification exams ensure the exchangeability with the intervention classes. In addition, we adjusted for potential confounders to estimate the true associations. Third, almost all (> 94%) recruits in each group consented to participate in the study, and more than 75% of them completed the study follow-up. The relatively high participation and follow-up rate minimize possible selection bias. Finally, we used well-defined and validated tools to examine the health outcomes, freeing the study from information bias.

However, there are some limitations to our study. One of them is that we cannot test the pure effects of a single intervention. In the current study, the outcome differences are due to the combined effects from the two interventions (i.e. a lifestyle intervention and the pandemic related training curriculum adjustments). Although the effects could be differentiated based on existing literature and knowledge, there may be synergism or other interactions between the two interventions, which prevents us from identifying the pure effects due to either one of the exposures. Second, the objective measurements for the intervention group are not available at academy C, reducing the sample size and associated statistical power for those outcomes. Additionally, as the intervention classes took place in the midst of the pandemic, there may be unmeasured confounding factors related to the pandemic, such as decreased physical activity during weekends and rest time, reduced social contacts, and less traveling during holidays. Finally, since the present study was designed to examine the combined effects of all HLS intervention measures, we could not tell which individual measures are the most effective.

## Conclusions

In conclusion, the present study shows fire recruits receiving a combination of a lifestyle intervention and COVID-19 related curriculum adjustments during academy training had more improvements than the historical control group with respect to HLS scores, blood pressure, and depressive symptoms. On the other hand, likely due to pandemic impacts on training, the intervention group experienced less improvement in body fat, push-ups, and pull-ups. In order to examine the real changes due to the intervention, a future study enrolling additional, post-pandemic academy classes that are more comparable to the historical control group and sustain a pure lifestyle intervention is warranted.

## Supplementary Information

Supplementary Information.

## Data Availability

The datasets used and/or analyzed in the current study are available from the corresponding author on reasonable request.
