# Supplementary material for: Effects of a healthy lifestyle intervention and COVID-19-adjusted training curriculum on firefighter recruits
Source: Sci Rep. 2022 Jun 23;12:10607. doi: 10.1038/s41598-022-10979-2 (PMC9226180; doi:10.1038/s41598-022-10979-2)
Supplement: Supplementary file 1 — Supplementary Information. [file 41598_2022_10979_MOESM1_ESM.docx]

**Supplemental Table 1.** Baseline demographics, body composition, physical fitness, and mental health of the recruits of the three classes at academy B

|  | **Historical Control** | **Pandemic interrupted Class** | **Intervention Class** | ***P* value comparing recruits of the three classes** |
| --- | --- | --- | --- | --- |
| **N** | 36 | 38 | 37 |  |
| **Age** | 28.8 ± 6.5 | 30.7 ± 7.0 | 29.1 ± 4.9 | 0.385 |
| **Male N (%)** | 34 (94.4%) | 34 (89.5%) | 37 (100.0%) | 0.141^a^ |
| **BMI (kg/m^2^)** | 26.5 ± 3.2 | 29.1 ± 4.6 | 28.5 ± 4.2 | 0.022 |
| **%BF** | 17.5 ± 5.6 | 28.7 ± 7.4 | 23.9 ± 7.2 | <0.001 |
| **Push-Ups** | 36.6 ± 13.8 | 28.5 ± 9.2 | 28.7 ± 10.9 | 0.003 |
| **Pull-Ups** | 5.7 ± 4.8 | 5.1 ± 5.3 | 6.7 ± 5.2 | 0.436 |
| **Run Time 1.5 miles (minutes)** | 11.7 ± 1.3 | 12.1 ± 2.1 | 12.3 ± 2.2 | 0.498 |
| **PREDIMED score** | 6.4 ± 2.3 | 7.4 ± 2.2 | 7.1 ± 2.2 | 0.182 |
| **MEDI-lifestyle score** | 4.1 ± 1.2 | 3.7 ± 1.2 | 4.0 ± 1.1 | 0.287 |
| **Beck Questionnaire** | 0 (0-0.25) | 0 (0-1) | 0 (0-1) | 0.619^b^ |
| **PCL Questionnaire** | 2 (0-4) | 3 (0-7) | 1.5 (0-6) | 0.648^b^ |
| **PHQ Score** | 0 (0-1.25) | 0 (0-3) | 0 (0-2.25) | 0.741^b^ |

Median (Q1-Q3) for Beck questionnaire, PCL questionnaire, and PHQ score. Mean ± SD for other variables except for Male.

BMI: Body Mass Index. %BF: Percent Body Fat. Beck: Beck Depression Inventory for Primary Care. PCL: Post-Traumatic Stress Disorder (PTSD) Questionnaire. PHQ: Patient Health Questionnaire.

^a^ Derived from Fisher’s exact test.

^b^ Derived from Kruskal-Wallis rank sum test.

**Supplemental Table 2.** Changes in recruits’ selected body composition and physical fitness measurements from baseline to graduation of the three classes at academy B

|  | **Historical Control** | **Pandemic interrupted Class** | **Intervention Class** | ***P* value comparing recruits of the three classes** |
| --- | --- | --- | --- | --- |
| **N** | 36 | 38 | 37 |  |
| **BMI (kg/m^2^)** | 0.01 ± 0.04 | -0.01 ± 0.05 | 0 ± 0.03 | 0.065 |
| **%BF** | -0.05 ± 0.27 | -0.11 ± 0.13 | 0.01 ± 0.15 | 0.020 |
| **Systolic blood pressure (mmHg)** | -6.0 ± 10.3 | NA | -7.2 ± 10.0 | 0.595 |
| **Push-Ups** | 9.7 ± 6.5 | 7.1 ± 4.6 | 8.2 ± 4.9 | 0.118 |
| **Run Time 1.5 miles (minutes)** | -0.9 ± 0.6 | 0.2 ± 1.5 | -1.0 ± 0.9 | <0.001 |
| **MEDI-lifestyle score** | 1 (-1 – 2) | NA | 0 (0 – 2) | 0.848^a^ |
| **Beck Questionnaire** | 0 (0 – 0) | NA | 0 (-1 – 0) | 0.058^a^ |

Median (Q1-Q3) for MEDI-lifestyle score and Beck questionnaire. Mean ± SD for other variables. BMI: Body Mass Index. %BF: Percent Body Fat. Beck: Beck Depression Inventory for Primary Care. NA: Not Available.

Absolute number changes for push-ups and run time. Percent changes for BMI and %BF.

^a^ Derived from Wilcoxon rank sum test with continuity correction.

**Supplemental Table 3.** Multivariable adjusted selected health profiles comparing the academy training effects on the pandemic interrupted class to the historical control class at academy B

| **Outcome** | **Predictor** | **Beta Coefficient** | **Standard Error** | ***P* value** |
| --- | --- | --- | --- | --- |
| **BMI (kg/m^2^)** | Time | 0.137 | 0.111 | 0.220 |
|  | Interrupted Class | -2.055 | 0.824 | 0.015 |
|  | Interrupted Class × Time | -0.327 | 0.155 | 0.036 |
| **%BF** | Time | -0.846 | 0.371 | 0.024 |
|  | Interrupted Class | 7.954 | 1.182 | <0.001 |
|  | Interrupted Class × Time | -0.727 | 0.517 | 0.162 |
| **Push-Ups** | Time | 4.847 | 0.465 | <0.001 |
|  | Interrupted Class | 2.119 | 3.659 | 0.564 |
|  | Interrupted Class × Time | -1.295 | 0.649 | 0.048 |
| **Run Time 1.5 miles (minutes)** | Time | -0.476 | 0.098 | <0.001 |
|  | Interrupted Class | -0.949 | 0.490 | 0.057 |
|  | Interrupted Class × Time | 0.574 | 0.135 | <0.001 |

BMI: Body Mass Index. %BF: Percent Body Fat.

Statistics derived from age, sex, baseline BMI, baseline percent body fat, and/or baseline pushup capacity adjusted mixed effects models.

**Supplemental Table 4.** Multivariable adjusted selected health profiles comparing the academy training effects on the Intervention class to the historical control class at academy B

| **Outcome** | **Predictor** | **Beta Coefficient** | **Standard Error** | ***P* value** |
| --- | --- | --- | --- | --- |
| **%BF** | Time | -0.85 | 0.36 | 0.019 |
|  | Intervention Class | 4.37 | 1.71 | 0.013 |
|  | Intervention Class × Time | 0.77 | 0.50 | 0.125 |
| **Systolic blood pressure** | Time | -2.99 | 0.85 | <0.001 |
|  | Intervention Class | -4.17 | 2.81 | 0.142 |
|  | Intervention Class × Time | -0.64 | 1.19 | 0.595 |
| **Push-Ups** | Time | 4.85 | 0.48 | <0.001 |
|  | Intervention Class | -2.19 | 3.20 | 0.495 |
|  | Intervention Class × Time | -0.75 | 0.67 | 0.261 |
| **MEDI-lifestyle score** | Time | 0.28 | 0.11 | 0.017 |
|  | Intervention Class | 0.26 | 0.38 | 0.503 |
|  | Intervention Class × Time | -0.01 | 0.16 | 0.963 |
| **Beck questionnaire** | Time | -0.01 | 0.09 | 0.880 |
|  | Intervention Class | 0.50 | 0.40 | 0.220 |
|  | Intervention Class × Time | -0.24 | 0.13 | 0.064 |

BMI: Body Mass Index. %BF: Percent Body Fat. Beck: Beck Depression Inventory for Primary Care.

Statistics derived from age, sex, baseline percent body fat, baseline pushup capacity, and/or baseline Beck Depression Inventory for Primary Care score adjusted mixed effects models.

**Supplemental Figure.** Box plots showing the distributions of recruits’ selected health profiles at baseline, mid-training, and academy graduation comparing a) the historical control class (n=36), b) the pandemic interrupted class (n=38), and c) the Intervention class (n=37).

A. Body Mass Index (BMI); B. Percent Body Fat; C. Push-ups; D. 1.5-mile running time. The plots were derived from the recruits whose data were available at each time point. Post-hoc paired analysis with Holm adjustments were demonstrated by *: P<0.05, **: P<0.01, ***: P<0.001, ****: P<0.0001.

| **A**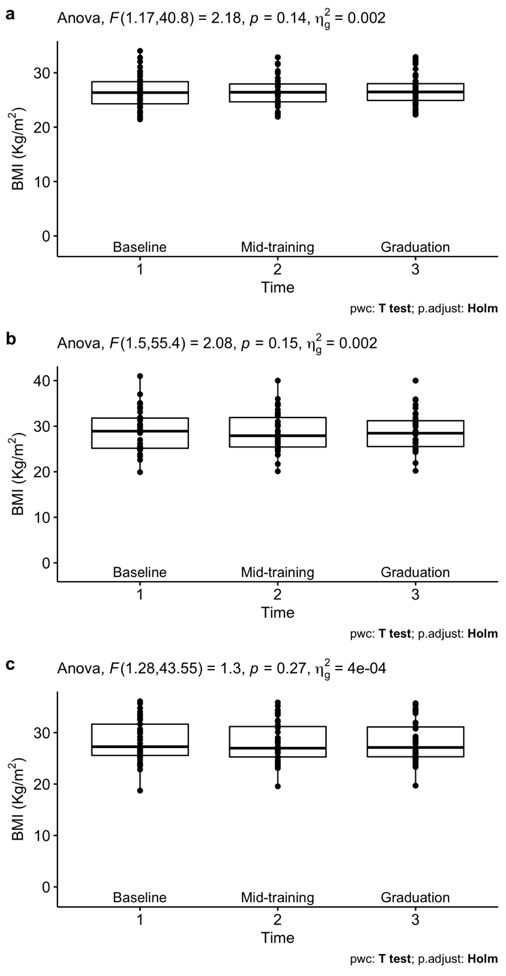 | **B**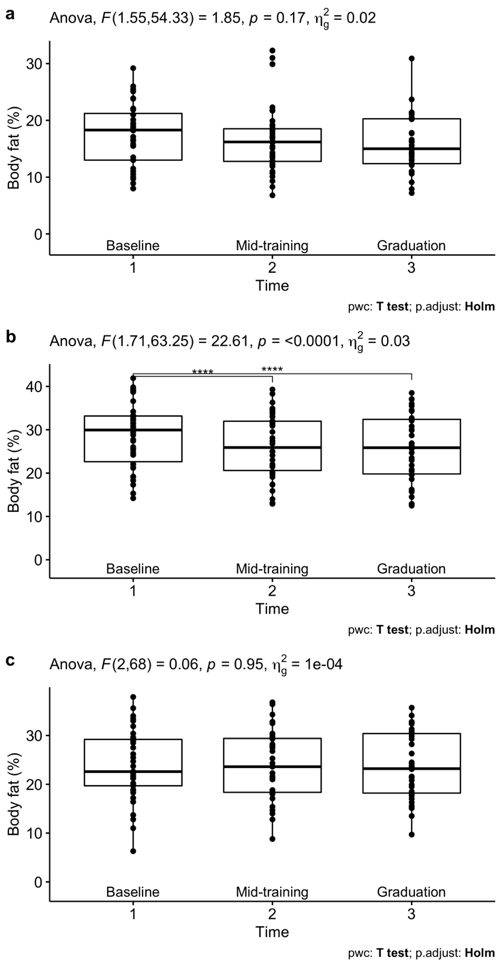 |
| --- | --- |
| **C**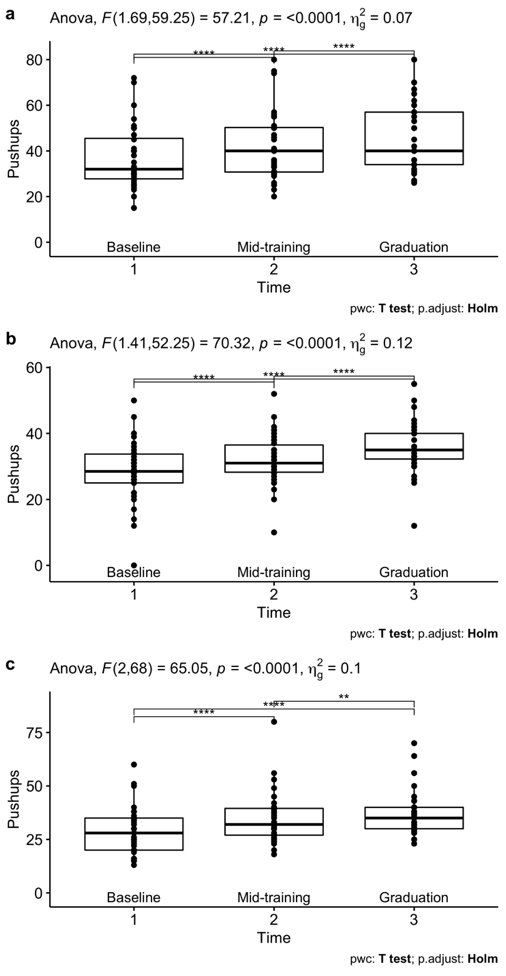 | **D**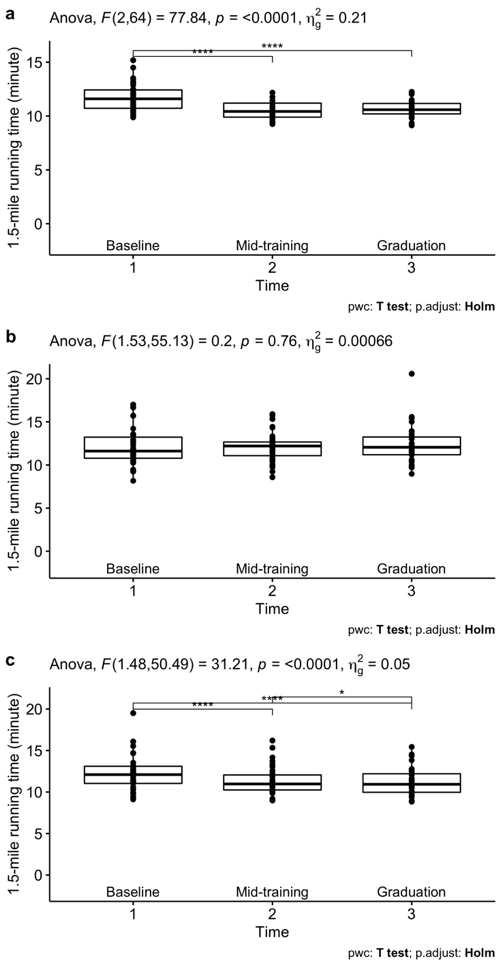 |
